# Dynamic QTL Analysis and Candidate Gene Mapping for Waterlogging Tolerance at Maize Seedling Stage

**DOI:** 10.1371/journal.pone.0079305

**Published:** 2013-11-14

**Authors:** Khalid A. Osman, Bin Tang, Yaping Wang, Juanhua Chen, Feng Yu, Liu Li, Xuesong Han, Zuxin Zhang, Jianbin Yan, Yonglian Zheng, Bing Yue, Fazhan Qiu

**Affiliations:** National Key Laboratory of Crop Genetic Improvement, Huazhong Agricultural University, Wuhan, China; Wake Forest University, United States of America

## Abstract

Soil waterlogging is one of the major abiotic stresses adversely affecting maize growth and yield. To identify dynamic expression of genes or quantitative trait loci (QTL), QTL associated with plant height, root length, root dry weight, shoot dry weight and total dry weight were identified via conditional analysis in a mixed linear model and inclusive composite interval mapping method at three respective periods under waterlogging and control conditions. A total of 13, 19 and 23 QTL were detected at stages 3D|0D (the period during 0–3 d of waterlogging), 6D|3D and 9D|6D, respectively. The effects of each QTL were moderate and distributed over nine chromosomes, singly explaining 4.14–18.88% of the phenotypic variation. Six QTL (*ph6-1, rl1-2, sdw4-1, sdw7-1, tdw4-1 and tdw7-1*) were identified at two consistent stages of seedling development, which could reflect a continuous expression of genes; the remaining QTL were detected at only one stage. Thus, expression of most QTL was influenced by the developmental status. In order to provide additional evidence regarding the role of corresponding genes in waterlogging tolerance, mapping of Expressed Sequence Tags markers and microRNAs were conducted. Seven candidate genes were observed to co-localize with the identified QTL on chromosomes 1, 4, 6, 7 and 9, and may be important candidate genes for waterlogging tolerance. These results are a good starting point for understanding the genetic basis for selectively expressing of QTL in different stress periods and the common genetic control mechanism of the co-localized traits.

## Introduction

Waterlogging is one of the most important constraints to maize production and productivity in tropical and subtropical regions around the world [1]. As the global climate is continuously changing, waterlogging is becoming a matter of prime importance for agricultural productivity and global food security [2]. In South-east Asia, about 15% of total maize growing area is affected by floods and waterlogging problem, causing 20–30% yield losses almost every year [1]. In China, there is a large area subjected to waterlogging at the maize seedling stage, especially in south-eastern China. In this area, heavy spring rainfall over a short period can lead to form the waterlogged soils for an extended period and cause severe damage to maize seedlings. Hence, new maize varieties with greater adaptation to waterlogging are essential to increase maize productivity in waterlogged soil. The development of waterlogging-tolerant varieties with high yield potential should be one of the main aims of many maize breeders [3]–[5].

With the development of DNA markers and quantitative trait locus (QTL) mapping methodologies, QTL analyses of waterlogging tolerance have been studied in several crops, such as rice [6]–[7], soybean [8], wheat [9] and barley [10]. Previous studies showed that the early stage of maize development is the most sensitive stage due to the growing point is below the soil surface during waterlogging time, especially from the second to the seventh leaf stage [4], [11]. Recently, several QTL mapping studies of waterlogging tolerance have been reported in maize and its wild relatives, *Z. luxurians* and *Z.nicaraguensis* [5], [12]. A larger number of QTL for waterlogging tolerance-related traits have been identified during the maize seedling stage, such as root and shoot development-associated traits [5], capacity for root aerenchyma formation [12]–[16], adventitious root formation [17]–[18], tolerance to toxins under reducing soil conditions and leaf injury [19].

To date, only two major QTL in rice, *Sub1A* [6] and *Snorkel* [7] have been map-based cloned, and these were found to encode ethylene-responsive factor-type transcription factors involved in gibberellin biosynthesis or signal transduction. Waterlogging tolerance of maize seedlings is a polygenic trait and is highly influenced by environment. Significant genotype by environment interaction could be detected by comparing QTL identified in multiple environments; and QTL with consistent expression across environments are required for breeding using marker-assisted selection (MAS) [20]. The stress effect in waterlogged soils is influenced by various environmental factors, including the degree and duration of stress, developmental stage of the plant, soil type and climatic circumstances.

In addition, the response of a plant to waterlogging can be conceptually divided into three stages with changes of gene expression occurring at different waterlogging periods [21]. The progress in developing cultivars with improved waterlogging-tolerance would be accelerated if the underlying genes could be identified. In our research group, a lot of candidate genes were observed by SSH (suppression subtractive hybridization) and cDNA microarray experiments [22]–[23]. MicroRNAs (miRNAs) are small non-coding RNAs that play critical roles in the regulation of gene expression at the post-transcriptional level. Recently, expression profiles of >100 expressed miRNAs changed in submerged maize roots were detected, and that submergence-responsive miRNAs might play a vital role in the regulation of metabolic, physiological and morphological adaptations [24]. Liu et al. [25] proposed a model of the regulation of differentially expressed miRNAs genes for sensitive, mildly-tolerant and tolerant inbred lines under short-term waterlogging conditions. The function of these candidate genes can be confirmed by mapping and co-localization with QTL, which may serve as additional evidence for the role of candidate genes in resistance (or defense response) to waterlogged conditions. The candidate genes can be used to develop functional markers for increasing selection efficiency in breeding programs [26]. Hence, mapping some of these candidate genes may offer a practical method for restricting the number of genes whose function should be validated [27].

Traits which change with time or with any other independent variable are important in agricultural research [28]. Up to now, almost all genetic studies of waterlogging tolerance have focused on crops at a specific or a final growth stage. Such studies could not fully capture the real gene action during plant growth and have neglected the developmental features of trait formation. A model to evaluate the net genetic effect (i.e. the conditional genetic effect) of a quantitative trait at a specific developmental stage was defined [29]. This genetic model was first applied in QTL analysis, and called conditional QTL mapping.

The conditional mapping method has been used to map QTL for plant height in maize [30], pod number and the main stem and plant height of soybean [31], seed weight of soybean [32], grain-filling rate in maize [33], plant height of wheat [34]–[35], linolenic acid content of soybean seed [36], root system architecture of maize [37] and resistance to late blight in potato [38]. However, so far, there has been no report on the use of conditional QTL mapping to reveal the dynamic analysis of QTL for waterlogging tolerance during maize seedling stages. We performed the present study with the aims to (1) identify the dynamic expression of QTL and novel genomic regions associated with waterlogging tolerance using conditional phenotypic values of maize seedling traits at different periods of 3, 6 and 9 d of waterlogging; (2) map some of the ESTs (expressed sequence tags) and the differentially expressed genes identified by SSH and microarray analysis and establish possible co-location between the candidate genes and QTL; and (3) analyze the relationships between candidate genes and mapped QTL for waterlogging-response traits. The conditional QTL analysis could map and estimate the net effect of waterlogging-related gene expression during different periods of waterlogging. This may provide better information to advance understanding of the genetic mechanisms of waterlogging tolerance, and develop elite maize lines with waterlogging tolerance through MAS.

## Materials and Methods

### Plant Materials and DNA Extraction

An F_2_ mapping population consisting of 247 F_2∶3_ lines were constructed from the cross between the waterlogging-tolerant maize line HZ32 and intolerant line K12. The two parents were selected based on their morphological and physiological criteria, including (plant height (PH), root length (RL), root dry weight (RDW), shoot dry weight (SDW), total dry weight (TDW = SDW+RDW) and the waterlogging tolerance coefficient) and (antioxidative enzymes and lipid peroxidation) respectively [11], [39]. The parents showed statistically significant differences for PH, RL, RDW, SDW and TDW under waterlogging conditions, but there were no differences under normal conditions [5], [11]. The F_2_ plants were used for genotyping SSR (simple sequence repeat) loci, and the seeds of the 247 F_2∶3_ lines derived from the corresponding F_2_ selfed-plants were utilized to conduct the waterlogging pot experiments. Total genomic DNA from F_2_ plants and two parental lines were isolated from young leaf tissue following a standard CTAB extraction method [40] with minor modifications.

### Pot Experiments and Phenotypic Measurements

The pot experiments were carried out under glasshouse conditions at Huazhong Agricultural University, Wuhan, China (114°36′E and 30°47′N) in 2010. The day/night temperatures were 33/17°C, with a photoperiod of 13/11 h. Three pot experiments were conducted in a randomized complete-block design with three replications. Two pots were included for each replication per genotype with one the control and the other the waterlogged treatment. Twelve seedlings per pot were included in each replication. The average values of 12 seedlings of the F_2∶3_ lines were considered to represent the phenotypes of the F_2_ plants. The seeds of the F_2∶3_ lines were planted in each pot of 20 cm in diameter and 30 cm in depth filled with 3.5 kg of sieved, sterilized dry field soil. The waterlogging treatments were conducted at the second leaf stage after 7 d of normal growth, and each pot was filled with 2–3 cm water above the soil surface and this water level was maintained until harvest. The controls were irrigated as needed to avoid drought stress or waterlogging stress. Twelve seedlings of each replication per genotype were harvested for trait scoring under waterlogged and control conditions at various waterlogging intervals (3, 6 and 9 d). After 3, 6 and 9 d of waterlogging stress, five waterlogging-related traits (PH, RL, RDW, SDW and TDW) of each replication per genotype under the waterlogging and control conditions were measured. The sampling, drying, and weighing methods were performed according to the previous methods [5]. PH was measured in a centimeter (cm) from the base of the culm to the tip of the longest leaf. RL was also measured in a centimeter (cm) from the base of the culm to the tip of the longest root. Roots and shoots of each pot were separately bulked together and put them into separate paper bags, respectively. SDW and RDW were measured at electronic balance (MP500B).

### Statistical Analysis

The best linear unbiased predictors (BLUP) for each family were computed using the ‘PROC MIXED’ procedure of SAS 8.02 [41] including treatment time (E) as a fixed effect, and genotype (G) or genotype by treatment time (G×E) interaction as random effects. For each trait, the normality of residual distributions was tested with skewness, kurtosis and the frequency distribution in the F_2∶3_ families were performed by Shapiro–Wilk test. Analyses of variance (ANOVAs) were performed using the general linear model (GLM) procedure of the SAS program. The broad sense heritability (*h^2^*) of seedling traits under waterlogged and controlling conditions was calculated based on each F_2∶3_ family mean from the three pot experiments with the following formula:

Where and are the estimates of genetic and residual variances, respectively, and n is the number of replications. Phenotypic Pearson’s correlations were calculated using the ‘PROC CORR’ option of the SAS program among four different seedling traits.

### Development of the Functional Markers and Simple Sequence Repeat (SSR) Genotyping

ESTs and miRNAs that were differentially expressed in response to short-term waterlogging at maize seedling stage [23], [25] were used to develop molecular markers. Design and primer sequences of EST markers used in this study were described previously [23]. Genome sequences of pre-miRNAs flanking 500 bp were analyzed with SSRIT to identify dinucleotide and trinucleotide motifs. A set of 110 locus-specific primers flanking the pre-miRNAs were designed using Primer 5. Primer sequences of 1052 SSR markers were obtained from the Maize Genetics and Genomics Database (www.maizegdb.org).

Polymerase chain reaction (PCR) amplifications of DNA markers were performed in a T1 Thermocycler Module 96 (Biometro, Goettingen, Germany). Each amplification reaction contained a volume of 20 µl, consisting of 6 µl of genomic DNA (10 ng/µl), 1.5 µl of MgCl_2_ (25 mM), 0.5 µl of dNTP mixtures (10 mM), 2 µl of 10×PCR buffer, 1.2 µl each primer pair (5 µM), 0.12 µl of Taq polymerase (5 units/µl) and 7.48 µl of double-distilled water. PCR parameters were as follows: 94°C for 5 min, and 31 cycles of 40 s at 94°C, 45 s at 58°C, 50 s at 72°C, then 5 min at 72°C. PCR products of the amplified DNA fragments were separated on 6% denatured polyacrylamide gel electrophoresis (PAGE) in 0.5×TBE buffer, followed by silver staining [5].

### Linkage Map Construction and Conditional QTL Mapping

**Figure 1:**
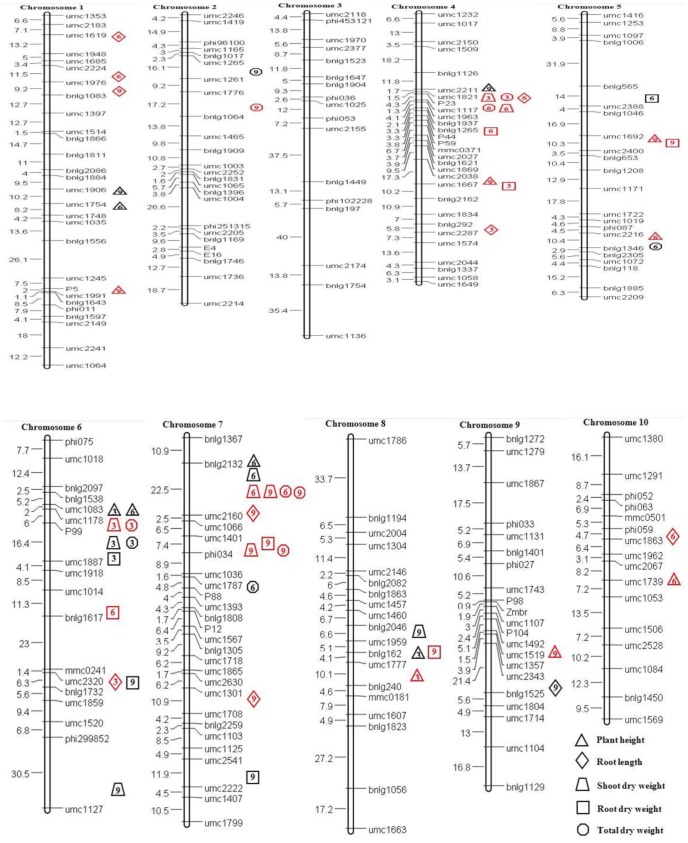
Positions of conditional QTL for five seedling morphological traits across three independent pot experiments (measured at the 3, 6 and 9 d of waterlogging) on linkage groups by ICIM. The number on the left side of each chromosome is the genetic distance between markers in cM. QTL are shown at the right side of each chromosome in different shapes for each trait and different colors for different treatments (black: control; red: waterlogging treatment).

Genotypic data of the F_2_ population were collected with 212 SSR, three EST and nine miRNA markers possessed clear and stable polymorphism in both parents. The χ^2^ test of F_2_ genotype against a 1∶2:1 segregation ratio was applied to identify markers with a distorted segregation and markers showing highly skewed segregation (*P*<0.001) were discarded. Molecular linkage maps were constructed using Mapmakers 3.0 [42] at a cutoff recombination fraction of 0.375, threshold logarithm of odds (LOD) score of 3.0 and the Kosambi function for estimation of map distances (cM). Finally, a set of 221 polymorphic markers between the parental lines on 10 linkage groups, spanned 1826.4 cM with an average distance of 8.15 cM between markers (Figure 1). Comparing them with the physical positions of the maize chromosome bin map, polymorphic markers resulted in coverage of 97 bins (except bins 1.12, 5.01 and 8.00), indicating the map had good coverage of maize’s 10 chromosomes. The linear order of SSR markers on the linkage map was in good agreement with previously published maize IBM 2008 neighbor’s maps, and no inversion in marker order was observed.

Conditional phenotypic values for each trait at time ‘t’ under different moisture regimes were calculated separately by subtracting the phenotypic means measured at time ‘t–1’ from the mean at time ‘t’ with software QGAStation 1.0 (http://ibi.zju.edu.cn/software/qga), in which the mixed model approach [29] was embedded. For the conditional QTL mapping, the single-environment QTL analysis was conducted on the conditional phenotypic values of each trait and each family at different 3 d time periods of waterlogging [3D|0D (i.e. the period of 0–3 d), 6D|3D and 9D|6D], using the method of ICIM-ADD for additive mapping [43]. The LOD thresholds for each trait of QTL were determined by 1000 permutation test at 95% confidence level and the walking speed for all QTL was 1 cM. For each trait; some marker intervals were revised if the position of multiple QTL peaks was <10 cM apart and regarded as a single QTL. Graphical representation of linkage maps was drawn using MapDraw v2-2 [44].

## Results

### Phenotypic Variation of F_2∶3_ Families

The contributions of genetic and environmental factors to several seedling traits observed under waterlogged and control conditions were determined by BLUPs for each trait in the F_2∶3_ families (Table 1). The values of PH, RL, SDW, RDW and TDW under waterlogging stress were significantly lower than those for controls. SDW and RDW under waterlogging stress showed nearly normal distribution at the threshold of *P*<0.01 (*W* = 0.99, *P* = 0.02 for SDW; *W* = 0.98, *P* = 0.01 for RDW), while other traits in the F_2∶3_ families fitted a normal distribution model (*P*>0.05). Both skew and kurtosis values of each trait were <1.0, suggesting that these traits were quantitative. Genetic variation was significant (*P*<0.05) for all traits investigated under different moisture regimes. ANOVAs showed highly significant difference in environmental variation (*P*<0.001) for all traits and most variations was due to treatment time of waterlogging, indicating strong environmental effects. Estimates of *h^2^* were relatively high for all five phenotypic traits across the three pot experiments, ranging from 0.66 for RL under a control condition to 0.85 for RDW and TDW under waterlogging stress. Comparing *h^2^* of all investigated traits showed that most of the measured traits under control conditions were always less than those calculated for phenotypic traits under waterlogging treatments. Significant positive associations (*P*<0.001) occurred among the PH, RL, SDW and RDW under waterlogging and control conditions (Table 2). The results indicated that these traits were not expressed independently of one another. As expected, the correlation coefficients were relatively higher between SDW and RDW. However, RL showed relatively low correlation coefficients with the SDW and RDW under both moisture conditions.

**Table 1:** Statistical analysis of the seedling morphological traits for 247 F_2∶3_ families across three independent pot experiments (measured at the 3, 6 and 9 d of waterlogging).

Conditions	Trait	Mean	Range	SD^a^	Skew	Kurt	W^b^	P^c^	G^d^	E^e^	h^2f^
Control	Plant height	27.26	22.00–34.14	2.15	0.15	−0.10	0.99	0.78	***	***	0.69
	Root length	33.58	29.99–36.55	1.26	−0.27	0.02	1.00	0.16	*	***	0.66
	Shoot dry weight	0.28	0.21–0.36	0.03	0.14	−0.03	0.99	0.59	***	***	0.73
	Root dry weight	0.17	0.11–0.23	0.02	−0.02	0.07	1.00	0.94	***	***	0.74
	Total dry weight	0.45	0.34–0.58	0.04	0.02	−0.08	0.99	0.53	***	***	0.73
Waterlogging	Plant height	22.70	17.15–29.33	2.19	0.09	−0.44	1.00	0.22	***	***	0.82
	Root length	16.90	12.82–21.05	1.49	0.02	−0.33	0.99	0.76	**	***	0.78
	Shoot dry weight	0.22	0.15–0.30	0.03	0.05	−0.66	0.99	0.02	***	***	0.84
	Root dry weight	0.06	0.04–0.09	0.01	0.32	−0.35	1.00	0.01	***	***	0.85
	Total dry weight	0.28	0.18–0.39	0.05	0.09	−0.62	0.98	0.07	***	***	0.85

a Standard deviation.

b w value of Shapiro-Wilk tests of Normality.

c P value of Shapiro-Wilk tests of Normality.

d–e ANOVAs results for the effect of F_2∶3_ families (G), treatment time (E). Differences between the mean values were significant at P<0.05 (*), 0.01 (**), 0.001 (***), or not significant (ns).

f The heritability was computed as:
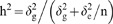

Where δ^2^_g_ and δ^2^_e_ were the estimates of genetic and residual variances and n was the number of replications.

**Table 2:** Simple correlation coefficients among traits measured in the F_2∶3_ families.

Conditions	Traits	Root length	Shoot dry weight	Root dry weight
Control	Plant height	0.4818***	0.5736***	0.4345***
	Root length		0.2857***	0.3670***
	Shoot dry weight			0.7552***
Waterlogging	Plant height	0.5595***	0.5529***	0.5077***
	Root length		0.4024***	0.4186***
	Shoot dry weight			0.7611***

Simple correlation coefficients were calculated between the traits using the adjusted means of the F_2∶3_ families across all environments.

The significance of correlation coefficient at P<0.05 (*), 0.01 (**), 0.001 (***).

### Genetic Mapping of Candidate Genes

A survey of 20 EST and 110 miRNA primer pairs identified three and nine loci polymorphic between the parents, using PCR-based markers corresponding to the candidate genes in response to waterlogging. Most candidate gene markers did not show polymorphism between the two parents in 6% PAGE, but the sequence analysis of these genes might help in developing single nucleotide polymorphism (SNP) markers in the future. Three EST markers were mapped on chromosomes 2 and 9. Nine miRNA markers were mapped on chromosomes 1, 4, 6, 7 and 9 through linkage analysis. P23, P88 and P104 had no similar sequence with micRNAs of known function in the maize database, but they shared high homology with other known sequences (Table 3).

**Table 3:** Map positions and primer sequences of candidate genes.

	Marker	Flanking marker	Bin	Forward primer (5′ to 3′)	Reverse primer (5′ to 3′)	Annotation
EST^a^	E4	bnlg1169–bnlg1746	2.08	ACCGGGATTCCCTCCGCCAA	TCACCGCCAGCTTGGCATCG	DNA-binding protein
	E16	bnlg1169–bnlg1746	2.08	GGTTGCTGGCTGCCTGGCTT	CCGTACGACGCTGGCTCACG	hypoxia induced protein
	Zmbr	umc1743–umc1107	9.04	CAACTCAAATAGCTGGT GGC	CCCGGTCAACCCTTGTTTTGTATG	DNA-binding protein
miRNA^b^	P5	umc1245–umc1991	1.08	CCGTGTTCTTTCTAAGTCGTT	ACATTCACGGTCAAGCAAC	zma-MIR166i
	P23	umc1821–umc1117	4.04	GTTGTTGTATTTCTCCGTCTCAC	GACTCAATCAATAGGCCCGAT	osa-MIRf11739-akr
	P44	bnlg1265–mmc0371	4.05	ACTTGTGACTATGAACCGAA	AGAATTGCCAAACTAGCTCT	zma-MIR319m
	P59	bnlg1265–mmc0371	4.05	CAGAGACTACATACGTGCTT	CCAATGCTTACATGCGTGA	zma-MIR319c
	P99	umc1178–umc1887	6.02	CTCACTCCTCTTCTGCTCGT	CCAGCAGCTACCTAATGCC	zma-MIR167i
	P88	umc1787–umc1393	7.02	GTTTGTACACGAGCCACGAT	TGTTAGACCTGATCATGAGCC	ptc-MIRf12019-akr
	P12	bnlg1808–umc1567	7.02	TTCAGTGCCATCCAACCCAG	GAAACAAAACCTCCAACGGTC	zma-MIR166j
	P98	umc1743–umc1107	9.04	ATAACTGAGCCTCACATGTCT	ACCCTGCAAAAGCCTCACG	zma-MIR169c
	P104	umc1107–umc1492	9.04	CTCGTTGATTTGCCAAGCTC	ACCAATCCAGCCTAACACC	mtr-MIR1510b

a Annotation analysis of ESTs was based on blastn and blastx at NCBI (http://www.ncbi.nlm.nih.gov/).

b Homology of identified miRNAs was obtained by searching in PMRD (http://bioinformatics.cau.edu.cn/PMRD/).

### QTL Detected for PH, SDW, RL, RDW and TDW

Considering conditional mapping for the five waterlogging-response traits by ICIM-ADD mapping, 13, 19 and 23 QTL were detected at stages 3D|0D, 6D|3D and 9D|6D, respectively (Table S1). Thirty-five and 20 QTL were detected under waterlogging treatment and control conditions, respectively. These QTL were distributed over all 10 chromosomes except for chromosome 3, and had LOD scores ranging from 3.05 to 12.32 explaining 4.14 to 18.88% phenotypic variation in the individual traits, which included 14, 10, 10, 10, and 11 QTL for PH, RL, SDW, RDW and TDW, respectively. The effect of individual QTL was generally small and 12 QTL individual accounted for more than 10% of phenotypic variance (Figure 1 and Table S1).

For PH, 14 QTL were identified under waterlogging treatment and control conditions on all 10 chromosomes except for chromosomes 2 and 3. Of seven QTL identified under waterlogging conditions, three and four favorable alleles were contributed by HZ32 and K12, respectively, individually explaining 4.49–18.88% of the phenotypic variation. For seven QTL under control conditions, alleles from HZ32 tended to increase the trait value and the individual contributions of these QTL were in the range of 4.14%–8.59%. One QTL (*ph6-1*) with the positive additive effects were detected at stage 3D|0D and 6D|3D, contributing 6.81 and 4.14% of the phenotypic variation, respectively.

Seven QTL for RL were detected on chromosomes 1, 4, 6, 7 and 10 under waterlogging treatment. Individual QTL explained 4.95–10.28% of the phenotypic variance and two QTL (*rl1-1* and *rl4-1*) with the negative additive effects were detected. One QTL (*rl1-2*) were detected at stages 6D|3D and 9D|6D, contributing 6.43 and 7.36% of the phenotypic variation, respectively. One QTL (*rl9-1*) was located on chromosome 9 under control conditions, accounting for 10.61% of the phenotypic variation. The allele from K12 tended to increase the RL.

Six and four putative QTL for SDW were detected under waterlogging and control conditions on chromosomes 4 and 6–8, respectively. Individual QTL accounted for 5.12–11.71% of the phenotypic variation. HZ32 contributed trait-enhancing alleles for 9 QTL and 1 QTL (*sdw7-2*) for K12 under waterlogging treatment. Only two QTL (*sdw6-1* and *sdw7-1*) were mapped in the same genomic region for both waterlogging and control conditions. Under waterlogging treatment, two QTL (*sdw4-1* and *sdw7-1*) were detected at two adjacent sampling intervals–their positive alleles were from HZ32 under both waterlogging and control conditions and both development stages, indicating that the same genetic elements may control gene expression.

Six and four QTL were significantly associated with RDW under waterlogging and control conditions, respectively. These QTL were located on chromosomes 4–8 and explained variances in the range of 5.07–10.01%. HZ32 alleles contributed to increase the RDW at six loci and K12 alleles contributed to increases at the other loci. No common QTL was found in both treatments and both sampling intervals.

There were seven QTL controlling TDW under waterlogging treatment, and the additive effects were positive at four loci. Under control conditions, four QTL were involved in TDW and the additive effects were positive at one locus. These QTL were mapped on chromosomes 2 and 4–7, which accounted for 6.95–11.46% of the phenotypic variance. One QTL (*tdw6-1*) was detected under both waterlogging and control conditions with a favorable allele from HZ32. In the waterlogging treatment, two QTL (*tdw4-1* and *tdw7-1*) were observed at two measuring stages. A conditional QTL (*tdw4-1*) with opposite genetic effects was detected at stages 3D|0D and 6D|3D, meaning that parental contribution of allele could change along with the time of waterlogging. One QTL (*tdw7-1*) had consistent genetic effects at stages 6D|3D and 9D|6D, and alleles from HZ32 tended to increase the trait values.

### Co-localization of QTL for different Traits

Taken together, about 87% of these QTL were co-located with at least one other QTL, forming eight QTL hotspots that controlled part of the variation for at least two different traits (Figure 1). The highest concentration of QTL was in the marker interval bnlg1126–umc117 on chromosome 4. Other impressive clusters of QTL were found on chromosomes 6 and 7. For example, one genomic region with QTL co-localized for more than three traits was detected on chromosomes 6 under control conditions. Three QTL hotspots were found on chromosomes 4 and 7 under waterlogging treatment, where QTL for more than three traits each was detected. The results indicated that these regions were under related genetic control. These traits all had highly significant positive associations with each other. The favorable alleles from most QTL clusters had the same direction of effect in all traits, but had opposite genetic effects at the other loci on chromosome 4.

### Co-localization of QTL and Waterlogging-responsive Genes

Co-locations of candidate genes and QTL for waterlogging-response traits could give additional evidence for the role of corresponding genes in resistance to waterlogging. To confirm their co-locations, 7 out of 12 candidate genes were genetically mapped within the confidence intervals of QTL for waterlogging-response traits on chromosomes 1, 4, 6, 7 and 9 (Table 4). Each QTL explained 4.57–18.88% of the phenotypic variance. Out of our expected to find that, five genes (*E4, E16, P12, P98 and Zmbr*) were not mapped to the QTL intervals for seedling traits.

**Table 4:** Co-location between mapped candidate genes and QTL for 5 waterlogging-response traits.

Marker	Chr.	Control					Waterlogging treatment				
		QTL^a^	Position^b^	LOD	A	R^2c^	QTL	Position	LOD	A	R^2^
P5	1						*ph1-3*	194	12.32	−2.268	18.88
P23	4	*ph4-1*	53	3.91	0.061	6.58	*rl4-1*	56	3.42	−0.103	4.95
							*sdw4-1*	55	3.09	0.008	5.12
							*sdw4-1*	59	3.91	0.002	6.35
							*tdw4-1*	55	3.93	0.014	7.03
							*tdw4-1*	58	5.47	−0.004	10.86
P44, P59	4						*rdw4-1*	71	4.49	−0.001	7.47
P99	6	*ph6-1*	28	3.97	1.616	6.81	*sdw6-1*	35	3.91	0.027	6.53
		*ph6-1*	28	4.73	1.239	5.59	*tdw6-1*	36	3.97	0.034	6.95
		*sdw6-1*	45	4.77	0.034	11.71					
		*tdw6-1*	46	4.21	0.046	10.02					
P88	7	*tdw7-2*	60	5.91	-0.034	7.08					
P104	9						*ph9-2*	83	3.26	−1.079	4.57

a For all QTL names, lowercase letter indicates traits abbreviations. ph = plant height; rl = root length; sdw = shoot dry weight; rdw = root dry weight; tdw = total dry weight. The first number following the letters represents the chromosome on which the QTL was located and the second number means the orders of the QTL located on the same chromosome by the same trait.

b Position of the peak of the QTL in centiMorgans.

c Percentage of the phenotypic variance explained by each putative QTL.

Under normal conditions, three genes (*P23, P99* and *P88*) had co-localized QTL for different traits with explained variances in the range of 5.59–11.71%. *P99* had co-localized QTL for the PH, SDW and TDW with the same direction of additive effects, and HZ32 alleles tended to increase the trait values.

Under waterlogging treatments, *P23* fell within a cluster of QTL for the RL, SDW and TDW with explained variances in the range of 4.95%–10.86%, but the alleles had opposite genetic effects across different traits or measured periods. *P99* was co-located with two QTL (*sdw6-1* and *tdw6-1*) and the QTL alleles had the same direction of effect. *P5, P44, P59* and *P104* were co-located with one QTL, respectively. The favorable alleles had the same direction of effect and the additive effects were negative in PH and RDW.

## Discussion

### Temporal Expression of QTL

Previous studies revealed considerable genotypic variation among different maize inbred lines in response to waterlogging [45]. In the present study, PH, RL, SDW, RDW and TDW were strongly inhibited by waterlogging stress and exhibited the typical distribution of quantitative traits within the population (Table S2), which was consistent with previous reports [5]. The best sustained treatment time for the evaluation of waterlogging tolerance at maize seedling stage was 6 d, but prolonged (from 2 to 12 d) waterlogging caused significant changes in WTCs [11]. The temporal regulation of the genes or QTL underlying waterlogging tolerance may be dynamically expressed at different treatment times. Dynamic expression of QTL at different stress periods was closely related to the genes of temporal and spatial expression of different developmental stages in maize, which reflected the locus differences of waterlogging tolerance. Analysis of conditional QTL at different treatment times will reveal the dynamic gene expressions, from which the net effect of a QTL at each time interval can be estimated [29]. Most QTL detected in 3D|0D, 6D|3D and 9D|6D stages differed, including locus positions, effect sizes and modes of action. No single QTL was continually active across three measured periods, whereas most QTL were active only at one or two time intervals in the population. This result indicated that different waterlogging stress times might induce different genes for tolerance and some genes might repeat their expression at different treatment stages. Similar results have also been observed in dynamic QTL mapping of rice blast resistance from seedling through to tillering and heading stages [46]. The number and effect sizes of QTL for waterlogging tolerance changed, with an increase from 3 to 9 d of waterlogging. The increased genetic effect with increased stress time has also been reported in QTL mapping for aluminum tolerance in rice, which could explain the higher aluminum tolerance in older seedlings [47]. As reported in conditional QTL mapping for plant height in rice [48], the QTL (*tdw4-1*) expressed effects in one direction at a certain stage but often later in the opposite direction. This suggests that the expression of some tolerant genes might express differently along with the stress time. These genes with opposite genetic effects expressed at the same or similar genomic positions might counteract each other. These may also explain why many conditional QTL were not detected in previous studies. Although most QTL were detected under one specific regime, three QTL (*sdw6-1, tdw6-1* and *sdw7-1*) were detected under waterlogging and control conditions, suggesting a constitutive rather than a stress responsive pattern of gene expression. This may also contribute to waterlogging tolerance, because it facilitates trait stability. The results are clearly in agreement with the developmental genetics theory in which the development of complex traits is environmentally dependent and dynamic through the actions and interactions of many genes [49]. Compared to previous studies that examined the same population only at 6 d of waterlogging, some major QTL might be neglected. These findings reveal the dynamic expression of QTL during the development of waterlogging-response traits at different stress periods. This may improve our understanding of the genetic control of maize waterlogging tolerance at the seedling stage and provide more information for MAS and map-based cloning of QTL.

### Comparisons with Previous Studies

Of the 55 QTL identified in this study, several important QTL clusters were localized on chromosome region bins 1.01, 4.03–4.05, 4.07–4.08, 5.03–5.04, 6.02–6.03, 7.00–7.01, 7.02 and 8.05 (Figure 1). The incidence of QTL clusters in similar genomic regions reflected trait associations [50], suggesting the possibility that the pleiotropic effects of single or closely linked genes might control plant development under waterlogging conditions and make an important contribution to enhancing tolerance to waterlogging. The QTL clusters could be deployed for improving waterlogging tolerance in maize through MAS. By comparing locations within chromosome bins of these QTL clusters, several major QTL for waterlogging tolerance-related traits that were identified in previous studies were near or the same chromosome regions in the present study. One major QTL (*ph1-3*) mapped to the umc1245–umc1991 interval on chromosome 1 were located near the region of a major QTL (*Qaer1.06*) for aerenchyma formation under non-flooded conditions [12]–[13], [15]–[16]. Using the same F_2_ population, most of the QTL identified by the waterlogging-response traits were also clustered in the chromosome region bins 4.03–4.05 and 7.02 [5]. Zhang et al. [45] identified a major QTL in bin 5.04 that controls waterlogging responses in PH and RDW using a genome-wide association study of 144 maize inbred lines. In the present study, the co-localized QTL for PH and RDW were also detected under waterlogging conditions in the marker interval umc1692-umc2400 on chromosome 5 (bin 5.03–5.04). A QTL cluster for PH and RDW located in the interval bnlg2046–umc1777 of chromosome 8 (bin 8.05) was found to share the same map location with a major locus for adventitious root formation under flooding conditions [17]–[18]. Previous studies have not reported QTL to be close to the major QTL or QTL clusters for waterlogging responses traits on chromosome region bins 1.01, 4.07–4.08, 6.02–6.03 and 7.00–7.01, indicating that these genomic regions may contain novel waterlogging-tolerance responsive genes. The result demonstrated that conditional QTL mapping for waterlogging responses traits in maize not only confirmed known waterlogging-tolerance loci, but also highlighted the utility of this method in mapping novel tolerance loci. With the increase of QTL numbers identified for waterlogging-response traits in different environments, the genetic basis of maize waterlogging tolerance will be become much clearer.

These QTL clusters may be very valuable for the simultaneous improvement of multiple traits, if favorable alleles at these loci originate from the same parent. Although common genetic mechanisms could exist for these traits, selection for beneficial alleles at all loci might be intricate due to the variability of various QTL effects. The detected favorable alleles had consistent effects in most QTL clusters for waterlogging-response traits, but the opposite genetic effects were observed at the other loci on chromosome region bin 4.04. It might be a reasonable interpretation that dynamics of QTL effects are likely due to selective gene expression at certain times [51]. According to a major QTL explaining >15% of the phenotypic variance in primary mapping [52], only one major QTL (*ph1-3*) with the negative effect was identified under waterlogging conditions in the present study (Table S1). This indicates that although accession K12 is phenotypically poor; it possesses some QTL alleles capable of increasing the trait value. Many previous studies have also found that QTL alleles enhancing a trait value originated from a phenotypically inferior parent in maize under various abiotic stresses, such as waterlogging [5], drought [53], low nitrogen [54], low phosphorus [55], and cold [56]. The results indicated that favorable alleles could derive from the waterlogging-sensitive parent and MAS to improve waterlogging tolerance should consider the effect directions and effect sizes of detected QTL at different durations of waterlogging.

In the present study, 43 of the 55 identified QTL individually accounted for <10% of phenotypic variance, but the overall proportion of phenotypic variance accounted for by the detected QTL was relatively low compared with the high heritability of the respective traits (Tables 1). The high *h^2^* implied that most of the phenotypic variance for each trait was genetic and could be effectively improved by selective breeding programs. These results suggest that the performance of quantitative traits may be affected by epistasis and QTL×environment interaction that explained a considerable portion of the total genotypic variances of the measured traits, which is in agreement with other reports [57]–[59]. However, the expression of waterlogging tolerance in maize is genetically complex and influenced by environmental factors and it is difficult to accurately estimate epistatic QTL and QTL×environment interaction effects in the present study, owing to lack of repeated waterlogging stress. Although several clusters of QTL for different traits were detected, it is still not possible to distinguish between pleiotropy and tight linkage of different polygenes and this requires much closer investigation due to its significance. Fine mapping and cloning of functional genes underlying the clusters of QTL identified in this study will aid understanding how the genes function and new maize varieties of waterlogging tolerant develop. We conclude that the clusters of QTL with small effects also play a significant role at the maize seedling stage in the response to waterlogging stress, although positional cloning of such loci will be difficult or impossible.

### Association between Candidate Gene and QTL

Comparison of the positions of candidate genes and QTL is a suitable strategy to investigate the molecular basis of quantitative traits [60]. To further understand the tolerance mechanism for survival under waterlogging stress, the co-localization between mapped QTL and candidate genes for waterlogging tolerance in maize seedlings were confirmed through a genetic mapping approach. To identify whether the waterlogging-responsive genes were localized in QTL intervals for all seedling traits, seven candidate markers controlling waterlogging-response traits were genetically mapped in QTL intervals on chromosome region bins 1.07–08, 4.03–4.05, 6.02–6.03, 7.02 and 9.04. As in the case of submergence tolerance QTL in rice, QTL hotspot regions may contain several transcription factors that regulate gene expression and confer enhanced tolerance to plants [6]. By comparing the positions of candidate gene and QTL, *zma-MIR166i*(*P5*)was found to co-localize with the peaks of the QTL *ph1-3*. The accumulation of *zma-miR166* under submergence stress could modulate hormone homeostasis via regulating transcripts of *HD-ZIP*, *ARF* and *GAMYB* and then trigger adventitious root formation and lateral root development [24]. The gene encoding *zma-MIR319m* (*P44*) and *zma-MIR319c* (*P59*) positioned within the marker interval bnlg1265–mmc0371 on chromosome 4, and *P44* were located in the peaks of the QTL *rdw4-1*. The miRNA-mediated gene regulated sub-network also showed that *miR319* may be an active participant in signal transduction at the early stage of hypoxic conditions [25]. An immediately apparent candidate gene encoding a homolog of osa-MIRf11739-akr (*P23*) underlying the clustered QTL for waterlogging tolerance in the interval umc1821–umc1117 of chromosome 4 were mapped in the peaks of QTL *rl4-1*, *sdw4-1* and *tdw4-1*. The targets of osa-MIRf11739-akr, type 2C protein phosphatases, were vitally involved in ABA signaling [61], that functioned to inhibit growth and regulate plant stress responses [62]. The accumulation of type 2C protein phosphatases in maize seedlings was induced at a late stage of waterlogging stress [23]. The gene encoding *zma-MIR167i* (*P99*), involved in regulation of maize crown roots’ development in response to waterlogging stress [63], was detected in the peaks of the QTL *sdw6-1* and *tdw6-1.* The homolog of ptc-MIRf12019-akr (P88) was mapped within the confidence intervals of QTL *tdw7-2* and close to QTL clusters on chromosome bin 7.02. The target of ptc-MIRf12019-akr, TFIIIA-type zinc finger protein was involved in plant development and abiotic stress responses and conferred multiple abiotic stress tolerances in transgenic rice [64]. The homolog of mtr-MIR1510b (P104) was mapped within the intervals of a QTL (*ph9-2)*. One target of mtr-MIR1510b, a RING/U-box superfamily protein, may play a role in plants in response to various environmental stresses [65]. In soybean, upregulation of an ATL-type RING finger protein might involve defense, directing proteolysis or modifying protein trafficking machinery under waterlogging stress [66]. These findings illustrate that mapping of differentially induced ESTs and miRNAs may be helpful as a first step toward identifying the key candidate genes underlying the QTL clusters for waterlogging-response traits. A functional marker developed from candidate genes may be an excellent candidate for use in molecular breeding for waterlogging tolerance in maize improvement, although the functions of the genes underlying the QTL need to be confirmed by developing near-isogenic lines, association analysis based on candidate gene sequencing or functional complementation analysis.

## Conclusions

Beyond the simple identification of QTL, this study is the first to use conditional analysis and an inclusive composite interval mapping method to dissect the net QTL expression of maize seedlings at various stress periods of waterlogging. We identified multiple QTL clusters affecting many traits on chromosomes 4, 6 and 7, suggesting that our approach was useful in elucidating the genetic mechanisms underlying maize waterlogging tolerance. Mapping of waterlogging-responsive ESTs and miRNAs may lead to identification of new candidate genes underlying the QTL clusters for traits of interest. Although the functions of these genes need to be confirmed through transgenic and association analysis, the strategy used in the current study is a good starting point for the discovery and mapping of waterlogging-responsive genes. The research results may provide new insight into the molecular basis of the waterlogging-stress response of maize seedlings and useful molecular markers for MAS.

## Supporting Information

Table S1 **Putative conditional QTL detected from the F_2∶3_ families for plant height (PH), root length (RL), shoot dry weight (SDW), root dry weight (RDW), total dry weight (TDW) measured at 3, 6 and 9 d of waterlogging.**
(DOC)

Table S2 **Phenotypic values of five seedling traits for 247 F_2∶3_ families at the 3, 6 and 9 d of waterlogging.**
(DOC)
