# Supplementary material for: Dynamic QTL Analysis and Candidate Gene Mapping for Waterlogging Tolerance at Maize Seedling Stage
Source: PLoS One. 2013 Nov 14;8(11):e79305. doi: 10.1371/journal.pone.0079305 (PMC3828346; doi:10.1371/journal.pone.0079305)
Supplement: Table S1 — Putative conditional QTL detected from the F2∶3 families for plant height (PH), root length (RL), shoot dry weight (SDW), root dry weight (RDW), total dry weight (TDW) measured at 3, 6 and 9 d of waterlogging. (DOC) [file pone.0079305.s001.doc]

**Table S1.** Putative conditional QTLs detected from the F_2:3_ lines for plant height, root length, shoot dry weight, root dry weight and total dry weight of plants measured at 3 sampling periods (3, 6 and 9 d of waterlogging).

| Trait | QTL ^a^ | Period | cM ^b^ | Marker interval | LOD | R^2 c^ (%) | Add ^d^ |
| --- | --- | --- | --- | --- | --- | --- | --- |
| Control |  |  |  |  |  |  |  |
| Plant height | *ph1-1* | 9D|6D | 122 | bnlg1884-umc1906 | 4.92 | 8.49 | 1.696 |
|  | *ph1-2* | 6D|3D | 136 | umc1754-umc1748 | 6.11 | 8.22 | 1.411 |
|  | *ph4-1* | 9D|6D | 53 | bnlg1126-umc2211 | 3.91 | 6.58 | 0.061 |
|  | *ph6-1* | 3D|0D | 28 | umc1083-umc1178 | 3.97 | 6.81 | 1.616 |
|  | *ph6-1* | 6D|3D | 28 | umc1083-umc1178 | 4.73 | 5.59 | 1.239 |
|  | *ph7-1* | 6D|3D | 10 | bnlg1367-bnlg2132 | 3.39 | 4.14 | 0.943 |
|  | *ph8-1* | 3D|0D | 92 | umc1959-bnlg162 | 4.99 | 8.59 | 1.947 |
| Root length | *rl9-1* | 9D|6D | 110 | umc2343-bnlg1525 | 5.05 | 10.61 | -2.677 |
| Shoot dry weight | *sdw6-1* | 3D|0D | 45 | P99-umc1887 | 4.77 | 11.71 | 0.034 |
|  | *sdw6-2* | 9D|6D | 155 | phi299852-umc1127 | 3.88 | 10.24 | 0.039 |
|  | *sdw7-1* | 6D|3D | 15 | bnlg2132-umc2160 | 3.15 | 6.36 | 0.017 |
|  | *sdw8-1* | 9D|6D | 85 | bnlg2046-umc1959 | 3.42 | 8.26 | 0.015 |
| Root dry weight | *rdw5-1* | 6D|3D | 56 | bnlg565-umc2388 | 3.16 | 7.66 | 0.019 |
|  | *rdw6-1* | 3D|0D | 52 | P99-umc1887 | 3.08 | 5.07 | 0.017 |
|  | *rdw6-3* | 9D|6D | 101 | umc2320-bnlg1732 | 3.41 | 6.22 | -0.012 |
|  | *rdw7-2* | 9D|6D | 146 | umc2541-umc2222 | 3.22 | 7.19 | 0.013 |
| Total dry weight | *tdw2-1* | 9D|6D | 39 | umc1265-umc1261 | 3.89 | 10.17 | -0.023 |
|  | *tdw5-1* | 6D|3D | 163 | umc2216-bnlg1346 | 5.04 | 7.72 | -0.006 |
|  | *tdw6-1* | 3D|0D | 46 | P99-umc1887 | 4.21 | 10.02 | 0.046 |
|  | *tdw7-2* | 6D|3D | 60 | umc1036-umc1787 | 5.91 | 7.08 | -0.034 |
| Waterlogging |  |  |  |  |  |  |  |
| Plant height | *ph1-3* | 9D|6D | 194 | P5-umc1991 | 12.32 | 18.88 | -2.268 |
|  | *ph4-1* | 9D|6D | 116 | umc2038-umc1667 | 5.46 | 9.20 | 1.458 |
|  | *ph5-1* | 9D|6D | 88 | umc1692-umc2400 | 4.30 | 7.00 | -0.473 |
|  | *ph5-2* | 6D|3D | 154 | umc2216-bnlg1346 | 3.17 | 5.93 | -1.505 |
|  | *ph8-2* | 3D|0D | 103 | umc1777-bnlg240 | 3.47 | 7.04 | 1.486 |
|  | *ph9-1* | 9D|6D | 83 | umc1492-umc1519 | 3.26 | 4.57 | -1.079 |
|  | *ph10-1* | 6D|3D | 61 | umc2067-umc1739 | 3.17 | 4.49 | 0.214 |
| Root length | *rl1-1* | 6D|3D | 14 | umc1619-umc1948 | 6.89 | 10.28 | -2.409 |
|  | *rl1-2* | 6D|3D | 40 | umc2224-umc1976 | 3.99 | 6.43 | 1.793 |
|  | *rl1-2* | 9D|6D | 47 | umc1976-bnlg1083 | 4.06 | 7.36 | 0.527 |
|  | *rl4-1* | 6D|3D | 56 | umc1821-P23 | 3.42 | 4.95 | -0.103 |
|  | *rl4-2* | 3D|0D | 153 | bnlg292-umc2287 | 3.79 | 7.40 | 0.659 |
|  | *rl6-1* | 3D|0D | 101 | umc2320-bnlg1732 | 4.15 | 8.20 | 1.933 |
|  | *rl7-1* | 9D|6D | 32 | bnlg2132-umc2160 | 4.87 | 9.70 | 0.959 |
|  | *rl7-2* | 9D|6D | 115 | umc1301-umc1708 | 3.57 | 8.28 | 0.292 |
|  | *rl10-1* | 6D|3D | 45 | umc1863-umc1962 | 3.30 | 4.97 | 0.270 |
| Shoot dry weight | *sdw4-1* | 3D|0D | 55 | umc1821-P23 | 3.09 | 5.12 | 0.008 |
|  | *sdw4-1* | 6D|3D | 59 | P23-umc1117 | 3.91 | 6.35 | 0.002 |
|  | *sdw6-1* | 3D|0D | 35 | umc1178-P99 | 3.91 | 6.53 | 0.027 |
|  | *sdw7-1* | 6D|3D | 25 | bnlg2132-umc2160 | 4.82 | 10.48 | 0.031 |
|  | *sdw7-1* | 9D|6D | 25 | bnlg2132-umc2160 | 3.54 | 10.45 | 0.048 |
|  | *sdw7-2* | 9D|6D | 50 | phi034-umc1036 | 3.92 | 7.70 | -0.003 |
| Root dry weight | *rdw4-1* | 6D|3D | 71 | bnlg1265-P44 | 4.49 | 7.47 | -0.001 |
|  | *rdw4-2* | 3D|0D | 119 | umc2038-umc1667 | 3.28 | 6.03 | -0.008 |
|  | *rdw5-2* | 9D|6D | 90 | umc1692-umc2400 | 3.90 | 9.93 | -0.002 |
|  | *rdw6-2* | 6D|3D | 76 | umc1014-bnlg1617 | 3.49 | 5.53 | 0.008 |
|  | *rdw7-1* | 9D|6D | 49 | umc1401-phi034 | 4.05 | 10.01 | 0.005 |
|  | *rdw8-1* | 9D|6D | 92 | umc1959-bnlg162 | 3.11 | 6.53 | 0.018 |
| Total dry weight | *tdw2-2* | 9D|6D | 64 | umc1776-bnlg1064 | 3.14 | 8.59 | -0.062 |
|  | *tdw4-1* | 3D|0D | 55 | umc1821-P23 | 3.93 | 7.03 | 0.014 |
|  | *tdw4-1* | 6D|3D | 58 | P23-umc1117 | 5.47 | 10.86 | -0.004 |
|  | *tdw6-1* | 3D|0D | 36 | P99-umc1887 | 3.97 | 6.95 | 0.034 |
|  | *tdw7-1* | 6D|3D | 25 | bnlg2132-umc2160 | 4.49 | 11.46 | 0.041 |
|  | *tdw7-1* | 9D|6D | 27 | bnlg2132-umc2160 | 3.05 | 8.88 | 0.059 |
|  | *tdw7-2* | 9D|6D | 50 | phi034-umc1036 | 3.64 | 7.68 | -0.005 |

^a^ For all QTL names, lowercase letter indicates traits abbreviations. ph=plant height; rl=root length; sdw=shoot dry weight; rdw=root dry weight; tdw=total dry weight. The ﬁrst number following the letters represents the chromosome on which the QTL was located and the second number means the orders of the QTL located on the same chromosome by the same trait.

^b^ Position of the peak of the QTL in centiMorgans.

^c^ Percentage of the phenotypic variance explained by each putative QTL.

^d^ Additive effect: positive value indicates that the tolerant parent (‘HZ32’) allele increases the trait value; negative value is just the opposite.
