# Supplementary material for: Dynamic QTL Analysis and Candidate Gene Mapping for Waterlogging Tolerance at Maize Seedling Stage
Source: PLoS One. 2013 Nov 14;8(11):e79305. doi: 10.1371/journal.pone.0079305 (PMC3828346; doi:10.1371/journal.pone.0079305)
Supplement: Table S2 — Phenotypic values of five seedling traits for 247 F2∶3 families at the 3, 6 and 9 d of waterlogging. (DOC) [file pone.0079305.s002.doc]

**Table S2.** Phenotypic values of the F_2:3_ families for plant height, root length, shoot dry weight, root dry weight and total dry of plants measured at 3 sampling periods (3, 6 and 9 d of waterlogging).

| Trait | Period | Mean | Minimum | Maximum | Standard deviation | Skew | Kurt |
| --- | --- | --- | --- | --- | --- | --- | --- |
| Control |  |  |  |  |  |  |  |
| Plant height | 3D|0D | 22.15 | 13.00 | 35.00 | 4.26 | 0.11 | -0.27 |
|  | 6D|3D | 27.12 | 16.84 | 39.05 | 3.89 | 0.26 | 0.22 |
|  | 9D|6D | 33.51 | 22.90 | 42.37 | 4.01 | -0.09 | -0.52 |
| Root length | 3D|0D | 27.76 | 11.75 | 46.00 | 5.63 | -0.06 | 0.35 |
|  | 6D|3D | 34.28 | 22.50 | 47.25 | 4.19 | 0.22 | 0.46 |
|  | 9D|6D | 39.17 | 26.00 | 55.20 | 5.78 | 0.15 | -0.48 |
| Shoot dry weight | 3D|0D | 0.22 | 0.10 | 0.37 | 0.06 | 0.33 | -0.49 |
|  | 6D|3D | 0.27 | 0.09 | 0.43 | 0.07 | 0.24 | -0.07 |
|  | 9D|6D | 0.36 | 0.19 | 0.61 | 0.09 | 0.26 | -0.49 |
| Root dry weight | 3D|0D | 0.12 | 0.04 | 0.24 | 0.04 | 0.35 | -0.02 |
|  | 6D|3D | 0.17 | 0.07 | 0.29 | 0.05 | 0.23 | -0.23 |
|  | 9D|6D | 0.21 | 0.11 | 0.37 | 0.05 | 0.32 | -0.06 |
| Total dry weight | 3D|0D | 0.35 | 0.16 | 0.57 | 0.09 | 0.25 | -0.49 |
|  | 6D|3D | 0.44 | 0.22 | 0.70 | 0.10 | 0.26 | -0.02 |
|  | 9D|6D | 0.57 | 0.30 | 0.92 | 0.13 | 0.13 | -0.60 |
| Waterlogging |  |  |  |  |  |  |  |
| Plant height | 3D|0D | 21.06 | 10.75 | 32.00 | 3.62 | 0.13 | 0.17 |
|  | 6D|3D | 22.22 | 9.42 | 32.62 | 3.90 | -0.10 | -0.13 |
|  | 9D|6D | 25.76 | 17.05 | 34.74 | 3.48 | 0.00 | -0.18 |
| Root length | 3D|0D | 18.62 | 8.00 | 33.00 | 4.52 | 0.05 | -0.28 |
|  | 6D|3D | 17.04 | 3.90 | 34.25 | 4.92 | 0.07 | 0.26 |
|  | 9D|6D | 14.98 | 8.93 | 20.90 | 2.19 | 0.08 | 0.12 |
| Shoot dry weight | 3D|0D | 0.21 | 0.04 | 0.38 | 0.07 | 0.23 | -0.27 |
|  | 6D|3D | 0.22 | 0.04 | 0.41 | 0.07 | 0.17 | -0.36 |
|  | 9D|6D | 0.23 | 0.08 | 0.42 | 0.07 | 0.13 | -0.31 |
| Root dry weight | 3D|0D | 0.05 | 0.01 | 0.13 | 0.02 | 0.56 | 0.40 |
|  | 6D|3D | 0.06 | 0.01 | 0.11 | 0.02 | 0.29 | -0.09 |
|  | 9D|6D | 0.07 | 0.02 | 0.13 | 0.02 | 0.38 | -0.26 |
| Total dry weight | 3D|0D | 0.26 | 0.06 | 0.47 | 0.08 | 0.20 | -0.32 |
|  | 6D|3D | 0.27 | 0.06 | 0.48 | 0.09 | 0.18 | -0.33 |
|  | 9D|6D | 0.30 | 0.10 | 0.55 | 0.09 | 0.14 | -0.29 |
